# A Matched Case-Control Study on the Association Between Colds, Depressive Symptoms during Pregnancy and Congenital Heart Disease in Northwestern China

**DOI:** 10.1038/s41598-018-36968-y

**Published:** 2019-01-24

**Authors:** Leqian Guo, Doudou Zhao, Ruo Zhang, Shanshan Li, Rong Liu, Hongli Wang, Shaonong Dang, Hong Yan

**Affiliations:** Department of Epidemiology and Biostatistics, School of Public Health, Xi’an Jiaotong University Health Science Center, Xi’an, Shaanxi 710061 China

## Abstract

The purpose of this study was to explore the association between colds, depressive symptoms during pregnancy and offspring congenital heart disease (CHD). A 1:2 matching case-control study was conducted in Northwest China. Information was gathered by a structured questionnaire and was reviewed by investigators on the spot. Multivariate logistic regressions and nonlinear mixed effect model were performed. 614 cases and 1228 controls were available in this study. After adjusting for potential confounders, the colds during the entire pregnancy were associated with increased risk of offspring CHD (OR = 1.44(1.12–1.85)). Similarly, there was a higher depression score in CHD group than the control group (OR = 1.89(1.48–2.41)). In addition, the women with both colds and higher depression scores had a higher risk of offspring CHD (OR = 2.72(1.87–3.93)) than their counterparts with only colds (OR = 1.48(1.04–2.09)) or with only higher depression scores (OR = 1.94(1.37–2.74)). The combined effects were significant in the multiplication model (OR = 2.04(1.47–2.83)) but not in the additive model (S = 1.40(0.70–2.81), AP = 0.19(−0.15–0.53) and RERI = 0.55(−0.54–1.64)). In conclusion, the colds and depressive symptoms during pregnancy were found associated with increased risk of offspring CHD and we found for the first time that there existed a statistically multiplying interaction effect of colds and depression on increasing risk of offspring CHD.

## Introduction

Congenital heart disease (CHD) referred to a cardiovascular malformation caused by abnormal cardiovascular development in the fetus. The prevalence of CHD was about 6.8 to 9.0 per 1000 live births worldwide^1–3^. It had become the most common form of human birth defects, accounting for nearly one-third of all major birth defects^2^. In addition, the CHD resulted in more than 40% of prenatal deaths^4^ and remained the leading cause of birth defect related to infant morbidity, mortality, and long-term disability^5^. It greatly affected the life quality of children and brought heavy medical burden to families and society^6^. In China, the prevalence of CHD was about 8 to 10 per 1000 live births^7^, which contributed a lot to the total number of CHD in the world because of the large population of China. Therefore, studying the control and prevention of CHD in Chinese population was not only beneficial to China but also provided evidence beyond China to some extent.

However, its underlying causes were still unclear^8^. Therefore, it was important to explore the possible etiological factors, especially where it could be modified, which may be a key step in the implementation of primary prevention. Over the past decade, a large number of epidemiological surveys had shown that a multitude of environment risk factors greatly contributed to the CHD. For example, maternal drinking, smoking, taking medicine and exposure to some physical factors during pregnancy were associated with the increased risk of offspring CHD^9,10^. On the other hand, pregnancy medical examination and taking folic acid may reduce the risk of offspring CHD^5^.

Increasing numbers of studies also had explored the association of various maternal illnesses, such as hypertension, diabetes mellitus, thyroid disorders and upper respiratory tract infection during pregnancy with CHD^11,12^. Due to changes in the mother’s internal environment during peri-conception, the body’s immune system will be reduced, and infectious diseases, such as cold and fever were more likely to occur^13,14^. There was growing evidence that maternal infection during pregnancy may be associated with spontaneous abortion, stillbirth and congenital defects in future generations^15,16^.

Physiological and psychological diseases often interact^17,18^. As the most common disease during pregnancy, the effect of colds on pregnant women’s mood was noteworthy. It was indicated that the prevalence of antenatal depression or anxiety during pregnancy was 7 to 11 per 100 pregnant women in high-income countries^19,20^, and was 11 to 27 per 100 in China^21^. Previous studies found maternal psychosocial stress during pregnancy was a possible risk factor for adverse birth outcomes^22–24^. For instance, the evidence from a population-based cohort study had shown that maternal psychosocial adversity during pregnancy was associated with offspring gestational age and birth weight^25^. It was also well established that maternal anxiety during pregnancy could result in offspring asthma^24^.

However, most studies had only reported one type of diseases, such as infectious diseases and febrile diseases instead of a specific disease, when analyzing the association between common diseases during pregnancy and offspring CHD. It was difficult to distinguish between the independent and joint effects between maternal colds and fever^10^. In terms of psychological factors, most studies focused on psychological stress and anxiety. Research on depression was mainly focused on postpartum depression or the association between depression and mother’s cardiovascular disease^26,27^. Other studies had included clinical trials of the efficacy of antidepressant drugs in psychiatric patients^28^. As far as we know, there was no specific epidemiological study on the association between depressive symptoms during pregnancy in the general population and offspring CHD. In addition, there was no article on exploring the interaction of colds and depressive symptoms during pregnancy on offspring CHD.

Therefore, this study focused on the association of common diseases during pregnancy that was colds and depressive symptoms, and offspring CHD. Because the prevalence of the CHD was increasing, we had reason to believe that even relatively small increases in effect would produce many cases. In addition, the combination of physiological factors and psychological factors followed the biopsychosocial model by Engel GL^29^. This study sought to better understand that association by assessing the interaction of colds, depressive symptoms on offspring CHD.

## Results

### Characteristics of the participants

**Figure 1:**
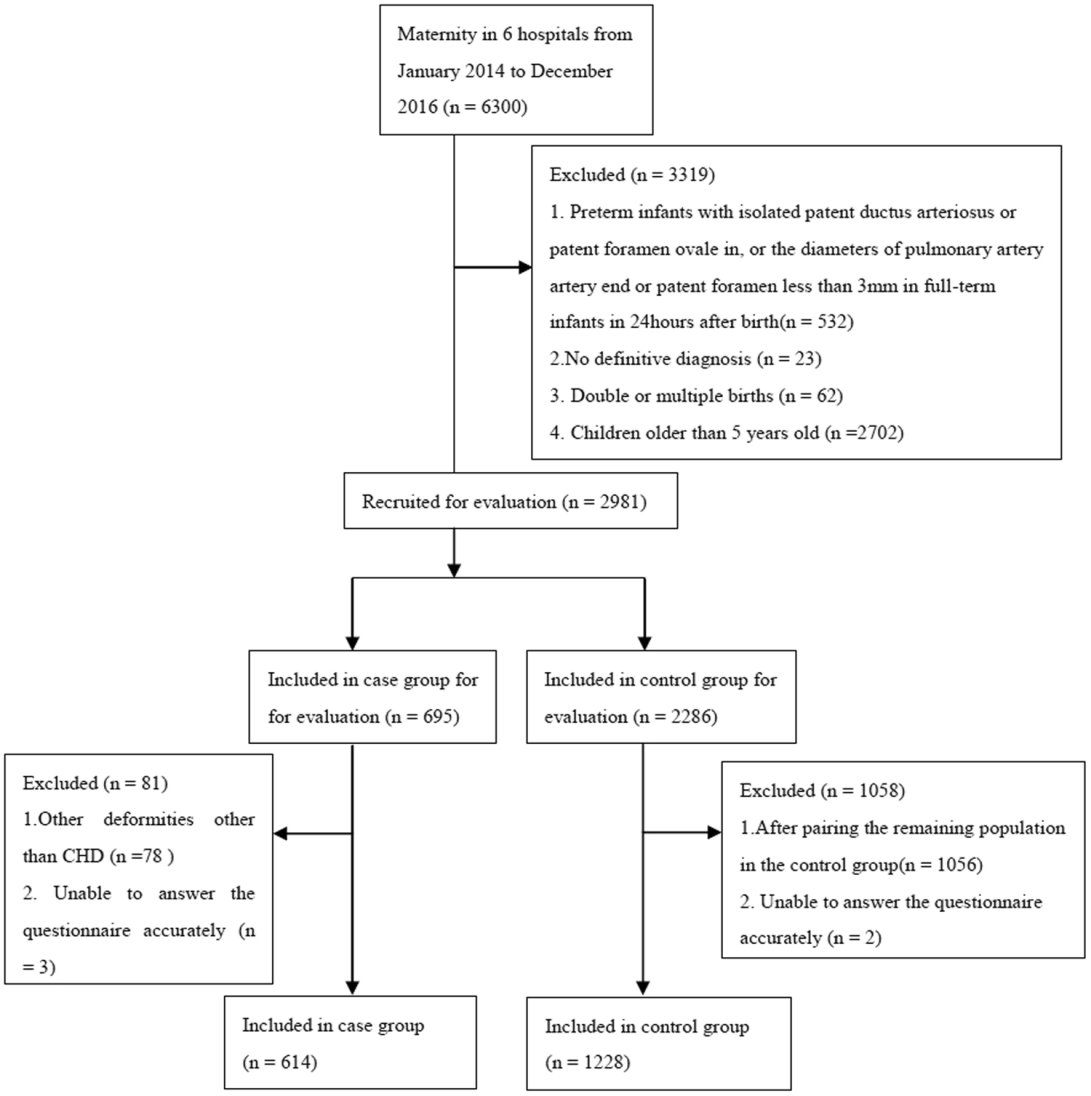
Flow-chart of study participants.

Flow-chart of study participants was shown in Fig. 1. 1842 subjects were available in this study, including 614 cases and 1228 controls. Maternal characteristics during pregnancy among infants with and without CHD were listed in Table 1. Compared with controls, case mothers were likely to more young, have lower education level, have a lower economic level, drink, take less folic acid, take medicine, have abnormal results of the prenatal examination, have negative life events and family history of offspring CHD (all *P* < 0.05). There were no statistical differences between two groups in maternal smoking, pregnancy reaction, chronic disease, natural abortion history and baby gender.

**Table 1:** Maternal characteristics during pregnancy among infants with and without CHD.

Characteristics	Controls	Cases	χ^2^ or fisher	*p*
**Age**				
<30	673(54.80)	453(73.78)	62.018	<0.001
≥30	555(45.20)	161(26.22)		
**Education**				
<Junior school	5(0.41)	36(5.86)	425.418	<0.001
Junior school	104(8.47)	168(27.36)		
High school	185(15.07)	244(39.74)		
>High school	934(76.06)	166(27.04)		
**Wealth index**				
Poor	238(19.38)	163(26.55)	15.321	0.001
Moderate	737(60.02)	355(57.82)		
Rich	253(20.60)	96(15.64)		
**Smoking**				
No	1224(99.67)	607(98.86)	—	0.030
Yes	4(0.33)	7(1.14)		
**Drinking**				
No	1220(99.35)	592(96.42)	21.958	<0.001
Yes	8(0.65)	22(3.58)		
**Taking folic acid**				
No	151(12.30)	113(18.40)	12.436	<0.001
Yes	1077(87.70)	501(81.60)		
**Taking medicine**				
No	884(71.99)	389(63.36)	14.287	<0.001
Yes	344(28.01)	225(36.64)		
**Pregnancy reaction**				
No	789(64.25)	378(61.56)	1.273	0.259
Yes	439(35.75)	236(38.44)		
**Abnormal prenatal examination**				
No	1135(92.43)	495(80.62)	56.037	<0.001
Yes	93(7.57)	119(19.38)		
**Negative life events**				
No	1089(88.68)	512(83.39)	10.085	0.002
Yes	139(11.32)	102(16.61)		
**Chronic disease**				
No	956(77.85)	495(80.62)	1.877	0.171
Yes	272(22.15)	119(19.38)		
**Natural abortion history**				
No	1144(93.16)	563(91.69)	1.295	0.255
Yes	84(6.84)	51(8.31)		
**Family history of CHD**				
No	1212(98.70)	594(96.74)	8.160	0.004
Yes	16(1.30)	20(3.26)		
**Baby gender**				
Male	647(52.69)	341(55.54)	1.337	0.248
Female	581(47.31)	273(44.46)		

### Association of congenital heart disease with colds or depressive symptoms

Table 2 showed the association between colds, depressive during pregnancy and offspring CHD. 319 (51.95%) cases and 560 (45.60%) controls were affected with colds during the entire pregnancy. Of these, 27 (4.40%) cases and 36 (2.93%) controls reported there was a fever when colds occurring. There were significant increases of CHD associated with colds during the entire pregnancy in Model 1, Model 2 and Model 3. The OR with 95% CI were 1.29(1.06–1.57), 1.35(1.05–1.73) and 1.44(1.12–1.85), respectively. Stratified analysis proved that the estimate between CHD risk and colds without fever was similar in Model 1, Model 2 and Model 3 (OR = 1.22(1.01–1.48), 1.27(1.01–1.63) and 1.35(1.05–1.73)). In addition, the ORs assessing colds in three months before pregnancy and the second trimester on CHD were 3.12(1.17–8.31) and 1.34(1.01–1.80) after adjusting for potential confounders (Model 3). The average depression scores of the cases and controls were 33.04 ± 6.28 points and 33.78 ± 5.26 points, respectively. There was a higher depression score in CHD group than the control group (*P* < 0.05) (Table 2). Only 49 (2.66%) subjects were classified as having depressive symptoms according to the cutoff value of 50. Finally, we divided it by 34 by calculating the Youden index, with 531 (43.24%) cases and 351 (57.17%) controls having the higher depression scores. Compared with continuous variables, classification variables could discover a higher risk in all models (OR = 1.75(1.44–2.13), 1.83(1.43–2.32) and 1.89(1.48–2.41)) (Table 2).

**Table 2:** Association between the offspring CHD and maternal exposure to colds and depressive symptoms during pregnancy.

Exposure	Controls	Cases	Model 1^a^	Model 2^b^	Model 3^c^
**Colds**					
No	668(54.4)	295(48.05)	1	1	1
Yes	560(45.6)	319(51.95)	1.29(1.06–1.57)	1.35(1.05–1.73)	1.44(1.12–1.85)
**Stratify according to fever**					
Colds with fever	36(2.93)	27(4.40)	1.52(0.92–2.53)	1.33(0.71–2.51)	1.38(0.73–2.61)
Colds without fever	525(42.75)	293(47.72)	1.22(1.01–1.48)	1.27(1.01–1.63)	1.35(1.05–1.73)
**Stratify according to exposure time**					
Three months before pregnancy	11(0.90)	13(2.12)	2.39(1.07–5.37)	3.19(1.18–8.64)	3.12(1.17–8.31)
Colds in first trimester	250(20.36)	146(23.78)	1.22(0.97–1.54)	1.12(0.84–1.5)	1.15(0.86–1.55)
Colds in second trimester	221(18.00)	144(23.45)	1.4(1.10–1.77)	1.27(0.95–1.71)	1.34(1.01–1.80)
Colds in third trimester	178(14.50)	56(9.12)	1.69(1.23–2.32)	1.33(0.91–1.95)	1.33(0.90–1.95)
**Depression scores**					
Continuous variable	33.04 ± 6.28	33.78 ± 5.26	1.02(1–1.04)	1.03(1.01–1.05)	1.03(1.01–1.05)
**Categorical variable**					
lower	697(56.76)	263(42.83)	1	1	1
higher	531(43.24)	351(57.17)	1.75(1.44–2.13)	1.83(1.43–2.32)	1.89(1.48–2.41)

^a^No other factors were adjusted.

^b^Adjusted for Model 1 and education, economic status, maternal smoking, drinking, taking folic acid, taking medicine, pregnancy reaction, abnormal prenatal examination, negative life events, chronic disease, natural abortion history, family history of CHD, baby gender.

^c^Adjusted for Model 2 and colds or/depression (continuous).

### Interactive effect analysis

Tables 3 and 4 evaluated the interactions of colds and depressive symptoms on offspring CHD. Table 3 showed 502 (27.25%) subjects caught a cold with lower depressive scores and 505 (27.42%) subjects only had a higher depression score without colds. There were 377 (20.47%) subjects with colds and higher depression scores. Compared with those who neither colds nor higher depression scores, the women with both colds and higher depression scores subjects had a significantly increased risk of offspring CHD (OR = 2.72(1.87–3.93)) after adjusting for potential confounders. In addition, the women with only colds and lower depressive scores had a significantly higher risk of offspring CHD (OR = 1.48(1.04–2.09)). Similarly, the women with only higher depression scores and no colds also had a significantly increased risk of offspring CHD (OR = 1.94(1.37–2.74)). When analyses were stratified by fever, the combined effects were significant for colds without fever (OR = 2.54(1.76–3.65)) but not for colds with fever (OR = 1.75(0.65–4.73)). Table 4 evaluated the interactions of colds and depressive symptoms by multiplication model and additive model. After adjusting for potential confounders, the variable of depression*colds had a significantly increased risk of offspring CHD (OR = 2.04(1.47–2.83)). Similarly, the variable of depression*colds without fever had a significantly increased risk of offspring CHD (OR = 2.21(1.57–3.09)). On the other hand, there was an increased risk of offspring CHD (S = 1.40(0.70–2.81), AP = 0.19(−0.15–0.53) and RERI = 0.55(−0.54–1.64)), but the differences did not reach the level of significance in the additive model (*P* > 0.05). When data were stratified by fever, there were still not statistically significant.

**Table 3:** Interactive effect (OR(95%CI)) of maternal colds and depressive symptoms on the occurrence of CHD.

	Depression^b^	N	Controls	Cases	Model 1^c^	Model 2^d^
**Colds** ^**a**^						
0	0	458(24.86)	358(29.15)	100(16.29)	1	1
0	1	505(27.42)	310(25.24)	195(31.76)	2.25(1.69–3.00)	1.94(1.37–2.74)
1	0	502(27.25)	339(27.61)	163(26.55)	1.72(1.29–2.30)	1.48(1.04–2.09)
1	1	377(20.47)	221(18.00)	156(25.41)	2.53(1.87–3.42)	2.72(1.87–3.93)
**Colds with fever** ^**a**^						
0	0	920(49.95)	674(54.89)	246(40.07)	1	1
0	1	859(46.63)	518(42.18)	341(55.54)	1.80(1.48–2.20)	1.88(1.47–2.40)
1	0	40(2.17)	23(1.87)	17(2.77)	2.03(1.06–3.85)	1.79(0.80–4.02)
1	1	23(1.25)	13(1.06)	10(1.63)	2.11(0.91–4.87)	1.75(0.65–4.73)
**Colds without fever** ^**a**^						
0	0	496(26.93)	380(30.94)	116(18.89)	1	1
0	1	528(28.66)	323(26.3)	205(33.39)	2.08(1.58–2.73)	1.81(1.30–2.52)
1	0	464(25.19)	317(25.81)	147(23.94)	1.52(1.14–2.02)	1.30(0.92–1.84)
1	1	354(19.22)	208(16.94)	146(23.78)	2.30(1.71–3.09)	2.54(1.76–3.65)

^a^0 = No and 1 = Yes.

^b^0 = lower score and 1 = higher score.

^c^No other factors were adjusted.

^d^Adjusted for Model 1 and maternal age, education, economic status, maternal smoking, drinking, taking folic acid, taking medicine, pregnancy reaction, abnormal prenatal examination, negative life events, chronic disease, natural abortion history, family history of CHD, baby gender.

**Table 4:** Multiplication models and additive models of maternal colds and depressive symptoms on the occurrence of CHD.

Interactions		Model 1^d^	Model 2^e^
Multiplication model	Depression * colds	2.08(1.58–2.74)	2.04(1.47–2.83)
Additive model	RERI^a^	0.59(−0.36–1.54)	0.55(−0.54–1.64)
	AP^b^	0.19(−0.09–0.47)	0.19(−0.15–0.53)
	S^c^	1.40(0.79–2.46)	1.40(0.70–2.81)
Multiplication model	Depression *colds with fever	1.55(0.67–3.55)	1.31(0.49–3.52)
Additive model	RERI	−0.72(−2.9–1.46)	−0.91(−3.18–1.35)
	AP	−0.34(−1.61–0.92)	−0.52(−2.23–1.18)
	S	0.61(0.11–3.39)	0.45(0.04–5.18)
Multiplication model	Depression *colds without fever	2.19(1.65–2.92)	2.21(1.57–3.09)
Additive model	RERI	0.78(−0.15–1.71)	0.83(−0.25–1.9)
	AP	0.26(−0.01–0.53)	0.28(−0.03–0.59)
	S	1.64(0.87–3.09)	1.75(0.8–3.85)

^a^The relative excess risk due to interaction.

^b^The attributable proportion.

^c^The synergy index.

^d^No other factors were adjusted.

^e^Adjusted for Model 1 and maternal age, education, economic status, maternal smoking, drinking, taking folic acid, taking medicine, pregnancy reaction, abnormal prenatal examination, negative life events, chronic disease, natural abortion history, family history of CHD, baby gender.

### Sensitivity analysis

Sensitivity analysis for interactive effects was based on a series of basic demographic characteristics. Tables 5 and 6 presented some of the results of sensitivity analysis. Compared with those who neither colds nor higher depression scores, both colds and higher depression scores subjects had significantly increased ORs of CHD among populations with different levels of economic activity, different levels of taking folic acid, and different baby sex (all *P* < 0.05). According to the ORs of the variable of depression*colds in Table 6, there were statistically multiplication effects in all subgroups (all *P* < 0.05). According to the S, AP and RERI, there were additive intersecting trends in all subgroups but the differences were not statistically significant (*P* > 0.05). The results of grouping by other demographic characteristics were similar, which were not shown in this article.

**Table 5:** Sensitivity analysis of interactive effect (OR(95%CI)) between maternal colds and depressive symptoms on CHD.

	Colds^a^	Depression^b^	N	Controls	Cases	Model 1^c^	Model 2^d^
**Wealth index**							
Poor	0	0	96(23.94)	70(29.41)	26(15.95)	1	1
	0	1	79(19.70)	45(18.91)	34(20.86)	2.03(1.08–3.83)	1.64(0.71–3.78)
	1	0	140(34.91)	84(35.29)	56(34.36)	1.80(1.02–3.15)	1.70(0.81–3.55)
	1	1	86(21.45)	39(16.39)	47(28.83)	3.25(1.75–6.02)	2.96(1.30–6.73)
Moderate	0	0	265(24.27)	206(27.95)	59(16.62)	1	1
	0	1	368(33.70)	222(30.12)	146(41.13)	2.30(1.61–3.28)	1.82(1.17–2.83)
	1	0	258(23.63)	185(25.1)	73(20.56)	1.38(0.93–2.05)	1.24(0.76–2.02)
	1	1	201(18.41)	124(16.82)	77(21.69)	2.17(1.45–3.25)	2.63(1.59–4.37)
Rich	0	0	97(27.79)	82(32.41)	15(15.63)	1	1
	0	1	58(16.62)	43(17.00)	15(15.63)	1.91(0.85–4.27)	1.89(0.75–4.76)
	1	0	104(29.80)	70(27.67)	34(35.42)	2.66(1.34–5.27)	2.40(1.10–5.21)
	1	1	90(25.79)	58(22.92)	32(33.33)	3.02(1.50–6.07)	3.43(1.55–7.58)
**Taking folic acid**							
No	0	0	66(25.00)	47(31.13)	19(16.81)	1	1
	0	1	50(18.94)	34(22.52)	16(14.16)	1.16(0.52–2.59)	1.01(0.34–3.01)
	1	0	76(28.79)	41(27.15)	35(30.97)	2.11(1.05–4.24)	3.22(1.25–8.32)
	1	1	72(27.27)	29(19.21)	43(38.05)	3.67(1.80–7.47)	4.22(1.53–11.64)
Yes	0	0	392(24.84)	311(28.88)	81(16.17)	1	1
	0	1	455(28.83)	276(25.63)	179(35.73)	2.49(1.83–3.39)	1.97(1.36–2.85)
	1	0	426(27.00)	298(27.67)	128(25.55)	1.65(1.20–2.27)	1.31(0.89–1.92)
	1	1	305(19.33)	192(17.83)	113(22.55)	2.26(1.61–3.17)	2.45(1.63–3.68)
**Baby gender**							
Male	0	0	243(24.60)	195(30.14)	48(14.08)	1	1
	0	1	275(27.83)	164(25.35)	111(32.55)	2.75(1.85–4.09)	2.30(1.42–3.72)
	1	0	261(26.42)	172(26.58)	89(26.1)	2.10(1.40–3.16)	1.82(1.12–2.96)
	1	1	209(21.15)	116(17.93)	93(27.27)	3.26(2.15–4.94)	3.50(2.11–5.82)
Female	0	0	215(25.18)	163(28.06)	52(19.05)	1	1
	0	1	230(26.93)	146(25.13)	84(30.77)	1.80(1.20–2.72)	1.65(0.99–2.76)
	1	0	241(28.22)	167(28.74)	74(27.11)	1.39(0.92–2.1)	1.24(0.74–2.08)
	1	1	168(19.67)	105(18.07)	63(23.08)	1.88(1.21–2.92)	2.16(1.24–3.76)

^a^0 = No and 1 = Yes.

^b^0 = lower score and 1 = higher score.

^c^No other factors were adjusted.

^d^Adjusted for Model 1 and maternal age, education, maternal smoking, drinking, taking medicine, pregnancy reaction, abnormal prenatal examination, negative life events, chronic disease, natural abortion history, family history of CHD and economic status/taking folic acid/baby gender.

**Table 6:** Sensitivity analysis of multiplication models and additive models between maternal colds and depressive symptoms on CHD.

Interactions			Model 1^d^	Mode 2^e^
**Wealth index**				
Poor	Multiplication model	Depression * colds	2.07(1.28–3.35)	2.01(1.06–3.83)
	Additive model	RERI^a^	0.42(−1.36–2.19)	0.62(−1.47–2.70)
		AP^b^	0.13(−0.39–0.65)	0.21(−0.43–0.85)
		S^c^	1.23(0.50–3.03)	1.46(0.37–5.77)
Moderate	Multiplication model	Depression * colds	1.37(1.01–1.88)	1.96(1.30–2.96)
	Additive model	RERI^a^	−0.51(−1.48–0.46)	0.57(−0.60–1.75)
		AP^b^	−0.23(−0.7–0.24)	0.22(−0.18–0.62)
		S^c^	0.7(0.36–1.33)	1.54(0.59–4)
Rich	Multiplication model	Depression * colds	1.68(1.01–2.82)	1.99(1.10–3.6)
	Additive model	RERI^a^	−0.55(−2.75–1.66)	0.14(−2.34–2.62)
		AP^b^	−0.18(−0.93–0.57)	0.04(−0.68–0.76)
		S^c^	0.79(0.32–1.94)	1.06(0.36–3.08)
**Taking folic acid**				
No	Multiplication model	Depression * colds	2.58(1.48–4.50)	2.43(1.10–5.39)
	Additive model	RERI^a^	1.39(−0.71–3.49)	0.99(−2.61–4.58)
		AP^b^	0.38(−0.08–0.84)	0.23(−0.51–0.98)
		S^c^	2.09(0.57–7.63)	1.44(0.37–5.66)
Yes	Multiplication model	Depression * colds	1.34(1.03–1.74)	1.77(1.28–2.45)
	Additive model	RERI^a^	−0.88(−1.79–0.03)	0.17(−0.77–1.11)
		AP^b^	−0.39(−0.82–0.04)	0.07(−0.30–0.45)
		S^c^	0.59(0.36–0.97)	1.14(0.56–2.31)
**Baby gender**				
Male	Multiplication model	Depression * colds	1.72(1.26–2.34)	2.13(1.43–3.17)
	Additive model	RERI^a^	−0.59(−1.93–0.74)	0.38(−1.16–1.93)
		AP^b^	−0.18(−0.61–0.24)	0.11(−0.31–0.53)
		S^c^	0.79(0.48–1.31)	1.18(0.60–2.34)
Female	Multiplication model	Depression * colds	1.36(0.96–1.93)	1.69(1.08–2.64)
	Additive model	RERI^a^	−0.31(−1.26–0.63)	0.27(−0.87–1.41)
		AP^b^	−0.17(−0.68–0.35)	0.12(−0.38–0.63)
		S^c^	0.74(0.31–1.75)	1.30(0.39–4.30)

^a^The relative excess risk due to interaction.

^b^The attributable proportion.

^c^The synergy index.

^d^No other factors were adjusted.

^e^Adjusted for Model 1 and maternal age, education, maternal smoking, drinking, taking medicine, pregnancy reaction, abnormal prenatal examination, negative life events, chronic disease, natural abortion history, family history of CHD and economic status/taking folic acid/baby gender.

## Discussion

Our findings showed that the colds during pregnancy were associated with increased risk of offspring CHD. Similarly, there was a higher depression score in CHD group. The women with both colds and higher depression scores had a higher risk of offspring CHD than their counterparts with only colds and lower depressive scores or with only higher depression scores and no colds. There was a statistically multiplying interaction effect of colds and depressive symptoms on increasing risk of offspring CHD. Stratified analysis also presented the stability of association of colds and depressive symptoms with CHD. These findings implied the importance of strengthening the guidance of pregnancy against the prevalence of offspring CHD.

After adjusting for potential confounders, we still observed a significantly increased risk of CHD with colds, which was in accordance with several studies. Oster *et al*. and Csaky-Szunyogh M *et al*. reported an association between maternal influenza during periconceptional period and a higher risk of offspring CHD^30,31^. Liu F *et al*. also reported that catching a cold was independent risk factors for isolated CHD^32^. The association was may stable in our study, because after stratification of fever and exposure time, the results showed the trends of increased risk except for colds with fever. Obviously, low incidence of fever caused by colds in our study could explain this situation. It was similar to previous evidence about exposure time. Liu F *et al*. indicated the maternal health condition, especially in the early trimester of pregnancy, was very important for the risk of heart defects^32^. There may be false exposure categories about three months before pregnancy and first trimester during pregnancy. The biological effects by which maternal colds with or without fever may result in heart defects were unclear. One of the most common assumptions was apoptosis, which was thought to be related to heart morphology and could be altered by fever and infection^33^.

In this study, depressive status in cases and controls were measured with SDS. There were several reasons for us to choose it. Firstly, previous studies had assessed the SDS as a valid and reliable instrument in identifying depression^21,34^. Secondly, we focused on early detection of depressive symptoms. The ZSDS score could not conduct clinically diagnose, but instead showed levels of depressive symptoms that might be clinically significant. In this study, due to the design of epidemiology, it was not possible to diagnose depression according to the clinical design standard manual. An easy, short-term, inexpensive self-report screening scale was optional. However, the results showed that most mothers were below the cutoff value of the scale itself, which may be due to small sample sizes and different populations. Of course, recall bias caused by investigation design was probably the most important reason. Moreover, individuals in China sometimes tended to be reluctant to show their negative emotions for fear of discrimination^35^.

The current study also showed that maternal depression scores, whether continuous or categorical, were associated with an increased risk of offspring CHD. Chou *et al*. observed a higher risk of offspring CHD in women with epilepsy and mood disorders during pregnancy^36^. Previous studies had suggested that stress during pregnancy were independently associated with increased risks of adverse birth outcomes^22,24^. In addition, perinatal depressive symptoms were associated with an increased risk of premature birth in a systematic review^37^. The mechanisms by which maternal depression may result in CHD remained unclear. It may be through hypothalamic-pituitary-adrenal axis hyperactivity and release of corticotrophin from the placenta. High levels of corticotropin-releasing hormone had been observed in relation to fetal growth restriction and birth defects^38,39^.

In the present study, we found for the first time that there existed a positive joint effect between maternal colds and depressive symptoms on the incidence of offspring CHD. The risk of CHD increased much more in women with both colds and higher depression scores subjects than those only having a cold or only having a higher depression score during pregnancy. Similarly, a positive combined effect still was found between maternal colds without fever and depression. Based on the interaction effect analysis, we found that the combined effect of the colds and depression could be multiplying effect. Sensitivity analysis indicated the stability of the results. Previous studies had examined the interaction between depression and other characteristics. For instance, it was well-established in some studies about the multiplying effect between smoking exposure and depressive symptoms on promoting atherosclerosis and heart disease^26,40^. Lou *et al*. found an additive effect of depression and nicotine addiction on the chronic obstructive pulmonary disease in a prospective cohort study^41^. In addition, a multiplying effect between memory disorders and depressive symptoms was observed to be associated exacerbate cognitive impairment in Alzheimer’s disease^42^.

The mechanism of interaction between colds and depressive symptoms for CHD may be complex and remain unclear. One hypothesis was that depression leads to a dysfunction in the anti-inflammatory response^43,44^. Some people had hypothesized that this inflammatory response explained the association between depression and cardiovascular diseases^45^. Colds were the most common type of respiratory infection and also increased systemic inflammation, which may explain the amplified association between colds, depressive symptoms, and offspring CHD. Closer interdisciplinary cooperation between physical and mental health care may help to determine possible common risk factors for depression and colds^46^. Future research should explore more potential mechanisms to further understand how colds and depression work together to increase the risk of offspring CHD.

This study tried to verify the association of offspring CHD with colds, depressive symptoms during pregnancy using a series of logical analysis of the data from the case-control study. Firstly, we established three logistic regression models in this study to investigate possible association before and after controlling for potential affecting factors. Then, the combined effect of colds and depressive symptoms was further explored by two methods, which were multiplication model and additive model, respectively. To our knowledge, ours was the first study to explore the interaction between pregnancy colds and depressive symptoms on offspring CHD. Further, we performed a series of sensitivity analyses to verify stability of this association. However, due to limitation of case-control study, we couldn’t draw causal inferences. In addition, there was an inconsistency between the cases and the controls, indicating the existence of selection bias. So we adjusted for possible covariates in our statistical analysis. Similarly, recall bias could have potentially affected our results because the interview was often based on parental recall after the birth of the child, especially when it came to assessing depression. Although controls were randomly matched at the rate of 1:2 by age of child, we may have missed some depressive symptom^40^. Moreover, colds and fever were largely determined by maternal self-reports, which it may lead to false exposure classification, and it was possible to miss a fever because women may be reluctant to disclose such information^47^. It may underestimate the effects of colds with fever during pregnancy and offspring CHD. Unfortunately, laboratory evidence or clinical diagnosis was difficult to obtain in retrospective studies. Finally, these findings come from only one study in China and need to be replicated in other ethnic groups.

In conclusion, our study found that the colds, depressive symptoms during pregnancy were associated with increased risk of offspring CHD, and we found for the first time that there existed a statistically multiplying interaction effect of colds and depression on increasing risk of CHD. These findings highlight the importance of considering pregnancy depressive status not only as potential risk factors for CHD but also in how the mental health may interact with physical health, which was valuable for pre-conception counseling and the identification of high-risk pregnant women. However, further studies of large samples are needed to confirm these results, to examine possible biological mechanisms, and to fully understand the effects of colds and depression on CHD through prospective studies.

## Methods

### Data and participants

The data used here was from a 1:2 matching case-control study with the purpose of exploring possible exposures in relation to offspring CHD. The study was conducted in Shaanxi province, Northwest China, which was a less developed region compared with Midwestern China. The study was carried out at 6 tertiary hospitals from January 2014 to December 2016: First Affiliated Hospital of Xi’an Jiaotong University, Xijing Hospital of Fourth Military Medical University, People’s Hospital of Shaanxi Province, Northwest Women’s and Children’s Hospital of Xi’an Jiaotong University, Second Affiliated Hospital of Xi’an Jiaotong University and Tangdu Hospital of Fourth Military Medical University, among which the first four hospitals were national birth defects monitoring hospitals. Ethics permission was approved by the ethics committee of the Xi’an Jiaotong University Health Science Center (No. 20120008). It was conducted in accordance with the relevant guidelines and regulations and we had obtained all participants’ written informed consents.

Cases were included based on the following inclusion criteria: termination of pregnancy lasted from January 2014 to December 2016; from 28 weeks after pregnancy to 7 days after birth, the perinatal children (including single live births and stillbirths) diagnosed with CHD according to the ICD-10 classification criteria, and pregnancy less than 28 weeks but diagnosed with CHD by ultrasound; exclude other deformities other than CHD. All birth defects were diagnosed by the doctors from the monitoring hospitals according to the relevant birth defect diagnostic criteria and classified according to the ICD-10 standard. Each classification had a corresponding diagnostic standard^48^. Controls were defined as singleton newborn infants without birth defects. At the same time, controls were randomly matched at the rate of 1:2 by age of child. The exclusion criteria included preterm infants (birth at less than 37 weeks gestation) with isolated patent ductus arteriosus or patent foramen ovale in, or the diameters of pulmonary artery end or patent foramen less than 3 mm in full-term infants in 24 hours after birth^32,49^. In addition, when the investigator explained the problem, parents who were unable to accurately answer the questionnaire due to mental illness or serious illness were also excluded.

From January 2014 to December 2016, Information was gathered by a structured questionnaire and was reviewed by investigators on the spot. Family data during pregnancy were reported by a face-to-face interview with mothers, including socio-demographic characteristics, lifestyles, environmental factors, disease and health conditions, nutrients and drug usage, physical examination, life events, pregnancy history and family history. Information on neonatal gender, weight, gestational age, birth outcomes and final diagnosis were collected by medical records at local hospitals. To minimize recall bias of exposure by mothers to the greatest extent, the interviews were conducted less than one year after the expected date of delivery. The questionnaire in this survey was designed by Xi’an Jiaotong University Health Science Center and modified according to a pilot study. All interviewed were conducted by well-trained doctoral and master students in epidemiology. The questionnaires and clinical diagnosis for all the cases and controls were examined by the experienced cardiovascular epidemiologists and obstetricians. We focused on exploring the association between maternal colds, depressive symptoms and offspring CHD in the present study. The datasets analyzed in the current paper were available from the corresponding author on reasonable request.

### Defining maternal colds and depressive symptoms

Data on maternal colds during different trimesters of pregnancy were gathered from mother’s self-report or through medical records if they sought medical care. Mothers were also asked to indicate whether fever had been present when they had a cold. According to the Chinese medical Guide^50^, the exposure to colds was divided into two types. One was the common cold, which was a mild viral infection of the upper respiratory tract (nose and throat). The other was influenza, generally more severe than the common cold. Influenza was an acute respiratory infection caused by the influenza virus, which included some additional symptoms such as fever, chills and muscle soreness. Fever referred to a pathological body temperature greater than 38 degrees.

For all cases and controls, the exposure time of depressive symptoms was defined as the entire pregnancy. Depressive symptoms were assessed with the developed Zung Self-rating Depression Scale (SDS)^51^. It was one of the most simple and common screening tools to discern subjective feelings of depression from nondepressive participants^52^. It had been well-validated and used in other Chinese studies^53^. The SDS was composed of 20 items covering somatic, affective and psychological features of depression. Every item had 1–4 scales. The standard score we ultimately need was obtained by multiplying the original total score by 1.25. The higher the score, the higher the likelihood of depression. More than 50 scores were considered to have moderate or strong symptoms of depression^51^. The Cronbach’s α of the SDS was 0.87. SDS was collected after subjects were interviewed. In this study, the Cronbach’s α of the SDS was 0.84 and Spearman-Brown Coefficient was 0.80. We examined depressive symptoms in both continuous and categorical form. For the categorical variable, there were two groups based on their standard total score: lower depression score group (<34) and higher depression score group (≥34). The cutoff value of 34 was determined by calculating the Youden index because the vast majority of people were in the subclinical stage in our study^54,55^.

### Covariates

Covariates in this study were selected on the basis of some evidences in the literature about examining the risk factors of CHD or other birth defects^10,32,36,39,49,56,57^. These covariates included maternal age (<30 and ≥30 years), education (<Junior school, Junior school, High school and >High school), economic status (Poor, Moderate, Rich), baby gender (Male or Female) and other variables classified into No or Yes, such as maternal smoking, drinking, taking folic acid, taking medicine, pregnancy reaction, abnormal prenatal examination, negative life events, chronic disease, natural abortion history and family history of CHD. Demographic and Health Survey wealth index was used to measure economic status, which was obtained by the principal component analysis^58^. Maternal smoking referred to smoking any cigarette and drinking was defined as drinking any liquor (>50 ml) at least one time during pregnancy. Taking folic acid referred to the folic acid supplement more than 30 days during pregnancy. Taking medicine was defined as taking any drugs during pregnancy, including antibiotics, anticancer drugs, antidepressants, hormones, hypoglycemic agents, antihypertensive drugs, salicylates. As for pregnancy reaction, mothers were asked whether there had been a severe early pregnancy reaction during pregnancy. Abnormal prenatal examination referred that normal B ultrasound, heart color ultrasound, four-dimensional B ultrasound or down’s screening had abnormal results. Negative life events were measured by maternal reports of the death of a close relative or friend, divorce or separation, poor living environment or working environment, relationship difficulties. In addition, chronic disease was defined as having any physical diseases during pregnancy, including gestational hypertension, hypotension, diabetes, hyperthyroidism, hypothyroidism, anemia, viral hepatitis and so on. All these exposures were asked based on the entire pregnancy.

### Statistical Analysis

The categorical variables were expressed as frequency and percentages. Depression score was expressed as mean ± SD. Chi-square test (*χ*^2^), Fisher exact test, and *t*-test were used for comparison between two groups. Multivariate logistic regressions were performed to investigate the association between colds, depression, and CHD controlling the confounders step by step. With CHD considered the dependent variable and colds or depressive symptoms considered the independent variable, respectively, we established three logistic regression models in this study: Model 1 wasn’t adjusted to any covariate. Model 2 was adjusted for all covariates (maternal age, education, economic status, maternal smoking, drinking, taking folic acid, taking medicine, pregnancy reaction, abnormal prenatal examination, negative life events, chronic disease, natural abortion history, family history of CHD and baby gender). Model 3 included all the outcome variables (colds and depressive symptoms) besides the covariates in Model 2. Similarly, stratified analysis of fever and exposure time was assessed using logistic regression.

The separate and combined effects of colds and depressive symptoms were also evaluated by logistic regression. The combined effect was further explored by two methods. On the one hand, we established the multiplication model by using logistic regression. We added the variable of colds*depressive (categorical) on the base of Model 2^59,60^. When odds ratio (OR) > 1, there was a positive biological multiplying effect. On the other hand, we used nonlinear mixed effect model method to establish the additive model^61^. The synergy index (S), the attributable proportion (AP), and relative excess risk due to interaction (RERI) were used to assess biological interactions in the additive model. S was the ratio of the combined effect to the sum of the individual effects. AP showed the proportion of the interaction effect in the total effect. RERI referred to the difference between the combined action of cold and depressive symptoms and the sum of individual effects. When S > 1, RERI > 0, or AP > 0, there was a positive biological additive effect. All analyses were adjusted for the covariates described above. In addition, we performed a series of sensitivity analyses that used the same approach based on three covariates: economic status, taking folic acid and baby gender.

All data were double-entered using EpiData version 3.1 (CDC, Atlanta, GA, USA). All statistical analyses were performed using SAS software, version 9.4 (SAS Institute) and were evaluated using 2-tailed 95% confidence intervals (CI). *P* < 0.05 was regarded as a statistic significance.
